# Update cerebellum and cognition

**DOI:** 10.1007/s00415-021-10486-w

**Published:** 2021-03-03

**Authors:** Heike Jacobi, Jennifer Faber, Dagmar Timmann, Thomas Klockgether

**Affiliations:** 1 Department of Neurology, University Hospital of Heidelberg, Heidelberg, Germany; 2 Department of Neurology, University Hospital of Bonn, Bonn, Germany; 3 German Center for Neurodegenerative Diseases (DZNE), Bonn, Germany; 4 Department of Neurology, Essen University Hospital, University of Duisburg-Essen, Essen, Germany

**Keywords:** Cognition, Cerebellum, Cerebellar cognitive affective syndrome (CCAS)

## Abstract

There is now robust evidence that the cerebellum—apart from its well-established role in motor control—is crucially involved in a wide spectrum of cognitive and affective functions. Clinical and neuropsychological studies together with evidence from anatomical studies and advanced neuroimaging have yielded significant insights into the specific features and clinical relevance of cerebellar involvement in normal cognition and mood.

## Introduction

The cerebellar cortex shows a complex and remarkably homogeneous cellular organization that is repeated across its entire extension. On the other hand, each part of the cerebellar cortex has specific feedforward and feedback connections to the cerebral cortex that go far beyond the long known connections to motor and premotor cortical areas. Indeed, there are parallel connections to paralimbic and association cortices. Based on these observations, it is thought that the cerebellum performs a universal transform that is applied to different domains, i.e., motor, cognitive and affective. According to current view, the cerebellum—based on its ability to detect errors in performing motor and mental processes—is building internal models, which are crucial for optimizing these processes and the basis for cerebellar learning across contexts [1, 2]. Through automation and adaptation, processes become increasingly stereotyped and efficient, and demand less attention [3]. Motor learning directly exemplifies the close link between the motor and cognitive domain of the cerebellum.

The various cerebellar functions are accordingly based on the specific anatomical connections of parts of the cerebellar cortex rather than on specific neuronal computations of each part. Consequently, a cerebellar functional topography can be defined that distinguishes motor, vestibular, cognitive, and limbic cerebellar regions. Whereas dysfunction of motor and vestibular parts are associated with ataxia, dysfunction of cognitive and limbic parts are associated with the cerebellar cognitive affective syndrome (CCAS) that was first described in the early 90s by Jeremy Schmahmann [4].

## Topography of cognitive functions in the cerebellum

The majority of the cerebellar cortex is not primarily involved in motor planning or execution. The motor-related areas of the cerebellum are relatively small and circumscribed in their extent and preserved across subjects [5–7]. According to the nomenclature introduced by Larsell (8), 10 cerebellar lobules are distinguished. Lobules I–V correspond to the anterior lobe, lobules VI–IX to the posterior lobe, and lobule X to the flocculonodular lobe. Lobule VII can be further subdivided in lobules Crus I, Crus II and VIIb. Crus I and Crus II together form lobule VIIa. Motor-related areas are located ipsilateral to the moved limb in lobules I–V with some extension into lobule VI and lobule VIII. In recent years, two approaches have largely been chosen to study the functional topography of the human cerebellum beyond sensorimotor processing: resting-state functional MRI (fMRI) for the assessment of cerebellar representation of cerebral networks and task-related functional MRI to directly identify the subsequent task-related areas.

Guell et al. [9] analyzed task fMRI data of the Human Connectome Project. Data from almost 800 human participants were included from a range of motor, working memory, language, social, and emotional tasks in the scanner. Language tasks activated primarily the right posterolateral cerebellar hemisphere, and spatial tasks primarily the left posterolateral cerebellar hemisphere. In the literature, however, lateralization of visuospatial functions is less clear within the cerebellum than language functions. Working memory, executive, social and emotional tasks were accompanied by activation of the posterolateral cerebellar hemispheres bilaterally [9]. In emotional tasks, activation was also found in the vermis. The vermis has known anatomical connections with the limbic system and has been referred to as “limbic cerebellum” [10]. Activations related to cognitive tasks were observed predominantly in lobule VII, with some extension into lobule VI. The most detailed map of motor and non-motor functions within the human cerebellar cortex to date has been published by King et al. in 2019. Their findings confirmed earlier observations that functional representations in the cerebellar cortex do not follow the boundaries of the cerebellar lobules. Furthermore, their findings indicate that the cerebellar cortex is organized in distinct functional subregions. King et al.’s findings are at variance with a recent resting-state fMRI analysis by Guell et al. [12] which suggests that two smooth gradients in the cerebellar cortex exist. According to this analysis, the main gradient extends from motor areas via non-motor task-focused (i.e., attentional/executive network-related) areas to non-motor task-unfocused (i.e., default-mode network related) areas. A second gradient extends from task-focused to task-unfocused non-motor areas [12]. Detailed maps of the functional topography of these non-motor functions based on task-based and resting-state fMRI data are publicly available and are helpful guides in neuroimaging and cerebellar lesion studies [11, 13].

Furthermore, resting-state data published by Buckner et al. [14] demonstrated a triple representation of cognitive functions in the cerebellar cortex, with two inverted representations in the posterolateral cerebellar hemispheres (attentual/executive network-related areas–default-mode network–default-mode network–attentual/executive network), and a third representation in lobules IX and X. The later overlaps with the well-known cerebellar–vestibular system. Findings were confirmed by Guell et al. [9] using task-related and resting-state fMRI data. The authors describe a triple non-motor representation in lobules VI/Crus I, Crus II/VIIB, and IX/X.

The concept of a non-motor role of the cerebellum is corroborated by cerebellar involvement in several psychiatric disorders, including autism [15, 16], schizophrenia [17, 18] and attention-deficit hyperactivity disorder [16, 19], as well as bipolar, major depressive and anxiety disorders [20]. The variety of psychiatric symptoms may be explained by projections of different cerebellar areas to specific extracerebellar areas.

## Cognitive and affective abnormalities in cerebellar patients

The cerebellum optimizes performance according to the context by modulating speed, capacity, appropriateness and consistency of cognitive and affective processes in a similar way, as it modulates force, rate, accuracy and rhythm of movements [21]. CCAS describes the salient features of cognitive and affective abnormalities in cerebellar patients [4, 22, 23]. Typical deficits can be grouped into four domains: deficits of executive functions, visuospatial cognition, linguistic functions and personality changes.

Cerebellar lesions in the posterior lobe of the cerebellum lead to deficits in executive functions that are similar to those seen with prefrontal lesions and include deficits in planning, sequencing, verbal fluency, working memory, abstract reasoning, problem solving strategies, set-shifting and an impaired ability to multi-task and organizing activities [4, 24]. Furthermore, such lesions can impair visuospatial and language functions. The latter may include agrammatism, dysprosodia, anomia, impaired syntax and deficits in verbal fluency [4, 24]. When damage affects the vermal and paravermal regions, patients show inappropriate behavior, mood disturbances including disinhibitions as well as regressive or compulsive behavior. With regard to neurobehavioral profiles, the following domains have been described: disorders of attentional and emotional control, deficits in social behavior as well as autism and psychosis spectrum disorders [21]. In the last few years, all aspects of the CCAS have been replicated in various populations of cerebellar patients in adults and children [24, 25].

In children, the first evidence of cognitive and affective abnormalities was derived from children after surgical resection of a posterior fossa tumors. Posterior fossa syndrome (PFS) is characterized by a reduction of speech or transient mutism, ataxia, hypotonia, executive dysfunction and affective and behavioral abnormalities [26]. Recovery may be prolonged and incomplete, showing residual coordination deficits, dysfluent and slowed speech as well as low social and educational achievements [27–29].

Early data about CCAS in adults mainly came from patients with focal injuries such as cerebellar strokes [4]. Typical motor abnormalities were shown in patients with lesions in the anterior lobe. When lesions spared the anterior lobe but affected lobules VI–X of the posterior lobe, patients showed minor or no motor impairment, but cognitive and affective abnormalities [30]. In detail, lesions leading to language deficits (e.g., right Crus I and II extending through lobule IX) could be distinguished from lesions resulting in visuospatial (bilateral Crus I, Crus II, and right lobule VIII) and executive deficits (lobules VII–VIII) [31]. Main predictors of recovery after cerebellar strokes are the localization, the cause and the extent of the lesion. Recovery from cerebellar stroke is better in ischemic stroke than hemorrhage, in strokes affecting the posterior and anterior inferior arteries than the superior cerebellar artery and in strokes leaving the cerebellar nuclei intact [32–34]. Additional extracerebellar involvement may complicate the functional recovery. Recovery from lesions of other etiology, e.g., multiple sclerosis seems to be worse [35].

The same pattern of deficits has been replicated in degenerative ataxias. Even before detailed genetic testing was available, impaired executive function was shown to be present in patients with dominantly inherited spinocerebellar ataxias. In a subgroup additional mild generalized cognitive impairment was present [36]. Since most subtypes of SCAs and other degenerative ataxias have significant extracerebellar pathology, studies of SCA6, are of great interest in furthering our understanding of cerebellar contribution to cognition because in this disorder the patients have an almost exclusive cerebellar pathology. Indeed, executive deficits involving inhibition of response and verbal reasoning, cognitive flexibility and abstraction have all been found in SCA6 [37, 38], whereas deficits seem to be more diverse and pronounced in multisystemic SCAs, such as SCA2 and SCA3 [37, 39]. Cognitive decline in SCA patients especially memory and learning abilities have been shown to deteriorate over time independently of motor functions or depressive symptoms [40].

## Clinical relevance and perspectives

Cerebellar involvement in cognition has long been a research topic but is gaining increasing clinical attention. Consideration of the CCAS in clinical routine is greatly facilitated by the recent availability of a validated bedside test that assesses the presence and severity of CCAS [23]. Thus, conventional bedside tests that were originally designed to measure cognitive and affective deficits in patients with dementia and depression are now dispensable. The CCAS scale allows screening of patients with cerebellar disease in a short time. Moreover, longitudinal assessment of cognitive and affective deficits in these patients is possible. One needs to be aware, however, that reference values which take age and education into account, are currently missing. The reference values published [23] may not apply for elderly patients [41].

Detailed knowledge of the presence, severity, and individual course of CCAS in patients with cerebellar disease is crucial because the presence of a CCAS may have an impact on the daily life of patients and their families due to cognitive and neuropsychiatric abnormalities, including inappropriate behavior or impulsive actions. The awareness of these symptoms provides the opportunity not only for better counselling, but also for active therapeutic intervention by cognitive rehabilitation, psychological treatment and in some cases pharmacological therapy of neuropsychiatric symptoms. In addition, the specific cognitive problems of cerebellar patients need to be taken into account by rehabilitation specialists. Rehabilitation programs for cerebellar patients that currently focus on coordination training and speech therapy need to be complemented by training programs that address the accompanying cognitive and affective symptoms. Such extended programs need to focus not only on executive and visuospatial functions and language, but also on psychological well-being and social behavior.

The cerebellum is increasingly considered as target for invasive and non-invasive neurostimulation, such as transcranial direct current stimulation (tDCS) and repetitive transcranial magnetic stimulation (rTMS). Current efforts are mainly focused on improvement of motor coordination in patients with cerebellar disease. However, it is conceivable that non-invasive stimulation could also be effective for improving cognitive function in these patients [42, 43]. However, due the highly convoluted nature of the cerebellar cortex, effects of non-invasive cerebellar brain stimulation are hard to predict, and robustness and replicability of previous findings will need to be seen before any recommendations on these types of therapy can be made [44].

Conversely, the understanding of the role of the cerebellum in cognition and affect could provide clues for the treatment of mental illnesses like autism-spectrum disorders, affective disorders or psychosis spectrum disorders as well as Alzheimer’s disease and frontotemporal dementia.
